# Supplementary material for: Cribriform plate microenvironment assembles a suppressive myeloid network during EAE-induced neuroinflammation
Source: eLife. 2026 Jul 8;15:RP110460. doi: 10.7554/eLife.110460 (PMC13345627; doi:10.7554/eLife.110460)
Supplement: Peer review history

# Editor report: eLife Assessment

**DOI:** 10.7554/eLife.110460.3.sa0

**Authors:** Isaac M Chiu

**Affiliations:** Harvard Medical School United States

**Keywords:** Convincing, Important

This **important** study identifies the cribriform plate as a key neuroimmune interface that shapes myeloid cell responses during neuroinflammation. Using imaging, flow cytometry, and single-cell approaches in a mouse model of EAE, the authors provide **convincing** evidence that dendritic cells and macrophages accumulate in PDPN-rich niches and have transcriptional features consistent with tolerogenic or immunosuppressive states. The work is technically strong and novel, and future studies will be needed to define the functional consequences of these myeloid cell states in autoimmunity.

# Referee report: Reviewer #1 (Public review)

**DOI:** 10.7554/eLife.110460.3.sa1

Summary:

Laaker et al. investigates the immunological role of the cribriform plate during neuroinflammation using the EAE model. The authors combine immunohistochemistry, flow cytometry and single-cell RNA sequencing to characterize CD11b+CD11c+ myeloid cells that accumulate at podoplanin (PDPN)-rich meningeal-lymphatic niches surrounding olfactory nerve bundles. They identified distinct populations of migratory dendritic cells (DCs) and macrophages retained at the cribriform plate that exhibit transcriptional signatures consistent with immune tolerance, reduced interferon signaling, and programmed cell death, including Pdcd1 (PD-1) expression. In parallel, CCR2+ monocytes and alternatively activated (M2-like) Arg1+/CHI3L3+ macrophages integrate into this niche, suggesting the establishment of a locally immunosuppressive myeloid network.

Strengths:

(1) Overall, the study postulates a novel model in which the cribriform plate functions as a specialized perineural immune interface that reshapes myeloid phenotypes during neuroinflammation.

(2) Suggests broader relevance for shaping peripheral immunity and therapeutic targeting. If DCs are being "tuned" at this exit site, it could influence what reaches cervical lymph nodes and how peripheral responses are set during CNS autoimmunity; the authors explicitly position this as relevant to CNS autoimmunity and possibly other CNS diseases (while acknowledging the need for human validation).

(3) Technical sound and highly original work. Convergent multi-method support: the central narrative is backed by immunohistochemistry + flow cytometry + scRNA-seq, rather than a single assay. The headline conclusion (tolerogenic/suppressive skew at the cribriform plate during EAE) is explicitly built from these combined modalities.

Comments on revised version.

All my points were adequately addressed by the authors.

# Referee report: Reviewer #2 (Public review)

**DOI:** 10.7554/eLife.110460.3.sa2

Summary:

In this article, Laaker et al described diverse populations of macrophages and dendritic cells found in and around the cribriform plate in a context of a neuroinflammation caused by an autoimmune disease (EAE). The authors utilize elegant histochemical staining and a nifty approach to sort doublets to interrogate cells that are in contact with one another, presumably in vivo. Notably, they uncover a population of CD11c+CD11b+ cells interacting with M2 macrophages and PDPN+ fibroblasts and lymphatics. These cells are heterogenous but some of these DCs express PD-1 and transcriptional profiling suggests they may have immunosuppressive behavior. Altogether, this article explains well the complexity of cell populations found around the cribriform plate during inflammation and are suggestive of different interactions that trigger these different phenotypes from immune cells.

Strengths:

Beautiful images of a unique CNS: peripheral interface that support a novel scRNA approach to understanding how different cell populations engage in functional interactions in vivo.

Weaknesses:

It is unclear how the sorted populations reflect in vivo interactions, or a propensity to form aggregates during ex vivo processing. Future work will be needed to address which poplanin expressing cells are most relevant.

# Author comment: Author response

**DOI:** 10.7554/eLife.110460.3.sa3

**Authors:** Collin Laaker, Martin Hsu, Andy Madrid, Jenna Port, Sophia M Vrba, Melinda Herbath, Cameron Baenen, Mohan Kumar, Thanthrige Thiunuwan Priyathilaka, Matyas Sandor, Zsuzsanna Fabry

**Affiliations:** University of Wisconsin-Madison Madison United States; University of North Carolina at Chapel Hill Chapel Hill United States; University of Wisconsin-Madison Madison United States; University of Wisconsin-Madison Madison United States; University of Wisconsin-Madison Madison United States; University of Wisconsin-Madison Madison United States; University of Wisconsin-Madison Madison United States; University of Wisconsin-Madison Madison United States; University of Wisconsin-Madison Madison United States; University of Wisconsin-Madison Madison United States; University of Wisconsin-Madison Madison United States

The following is the authors’ response to the original reviews.

**Reviewer #1 (Public review):**

(1) (Figure 1): Quantification of CSF1R-GFP^+^ and CD11c-eYFP^+^ cells in PDPN^+^LYVE-1^-^ vs. PDPN^+^LYVE-1^+^ regions. “This would demonstrate selective accumulation or retention of myeloid cells at the cribriform plate niche."

We thank the reviewer for this important suggestion. The representative images in Figure 1(Bottom) establish the partial justification for the cell sorting and sequencing strategy in Figure 3, which relies heavily on myeloid cells in contact with PDPN. Importantly, our previous publication Hsu et al. 2022 has quantified elevated Cd11c+ cells in contact with the Cribriform lymphatic niche. Figure 1 in this context seeks to show PDPN as an additional and broader marker for the meningeal and lymphatic tissue at the brain's border. Because PDPN represents more surface area, PDPN+Lyve-1- regions would likely show more immune cell accumulation but our primary argument is simply that myeloid cells also accumulate in both PDPN regions. As a result we argue the quantification of cells in Lyve-1+ and negative regions is not necessary. We have added a sentence to the text which explains the intention of the figure.

“Additionally, while PDPN labels the cribriform plate lymphatic vasculature, it also defines the meningeal-immune interface at the border of both the olfactory bulb and olfactory nerve bundles.”

(2) While the PostContact-seq strategy is innovative (Figure 3), additional justification is needed to demonstrate that tissue dissociation did not artificially disrupt PDPN-myeloid contacts. The relatively small proportion of live PDPN-rich doublets (~2.5% total aggregates and ~18% PDPN+ within total aggregates) raises questions about representativeness compared with in situ observations. The authors should also more explicitly elaborate on why PostContact-seq was favored over alternative approaches such as PIC-seq.

We acknowledge this important methodological concern. We have expanded the Methods section and added a dedicated paragraph in Results addressing the following:

Tissue dissociation controls: Dissociation protocols were used specifically to minimize cell-cell adhesion. Unfortunately, we cannot perform parallel dissociations of naive (non-EAE) cribriform plates for scRNAseq because PDPN^+^-containing doublets are essentially non-existent. This is also supporting the representativeness compared to in situ observation. Doublets are significantly enriched in EAE tissue compared to naive controls, arguing against artifactual aggregate formation.

Representativeness of ~2.5% doublets: While the absolute proportion of doublets is modest, this is consistent with in situ observations where myeloid-PDPN contacts are spatially restricted to the outer perineural and meningeal niche rather than globally distributed. We argue this is simply the enrichment of a rare interaction rather than a limitation.

PostContact-seq vs. PIC-seq: “PIC-seq (Giladi et al., 2020) sequences intact doublets and relies on specialized deconvolution tools to parse apart data. PostContact-seq leverages the cellular contact signatures post-dissociation, making it a more accessible system. However, we now explicitly discuss this comparison in the Results and acknowledge PIC-seq as a complementary future approach in discussion.

(3) (Figure 4B): Clarification of integration across four methods; consideration of CellChat/NicheNet: The authors stated that results regarding cell-cell interactions were integrated across four intercellular communication methodologies (Figure 4B), but this integration is not clearly described in either the Results or Method sections. This needs clarification. Moreover, the interaction analysis in Figure 4B seems to rely on TALKIEN, which does not incorporate prior ligand-receptor knowledge. Given the availability of widely used tools, such as CellChat and NicheNet, the authors may consider cross-referencing their findings.

We have revised the Methods sections to clearly describe our strategy for the TALKIEN analysis. Importantly, TALKIEN does integrate ligand receptor libraries from four sources: CellChat, CellPhoneDB, iCellNet, and the Ramilowsky datasets to generate its figures. Interactions reported in Figure 4B are those supported by this analysis, and we updated the text accordingly.

(4) Pseudotime trajectory analysis of CCR2^+^ monocyte differentiation.

"A pseudotime trajectory analysis may be valuable to test whether CCR2^+^ monocytes preferentially differentiate into CHI3L3^+^ macrophages, PD-1^+^ DCs, or other subsets."

We thank the reviewer for this insightful suggestion. We added a complete pseudotime analysis (Author response image 1). The pseudotime analysis tracks a continuous developmental trajectory starting from cDC2 and early macrophage populations (Pseudotime = 0, dark purple) and progressing through the main macrophage body toward an activated terminal state (Pseudotime = 16, yellow). Crucially, the trajectory correctly excludes non-continuous lineages such as resident microglia and lymphoid cells. This progression is functionally validated by the transient upregulation of the recruitment marker CCR2 during intermediate stages, which subsequently downregulates as cells transition into a mature phenotype.

**Author response image 1.**

(5) FACS-based validation of macrophage immunosuppressive signatures.

"Validation using the same post-contact vs. no-contact sorting strategy would strengthen the conclusions."

This is an excellent suggestion and will be the topic of future detailed investigation focusing on the cellular and molecular reprograming of the immunosuppressive microenvironment at the cribriform plate.

(6) Identity of CD45IV^+^ cells in contact with PDPN^+^ cells (Figure 6B-C); gating strategy; tissue co-labeling. "Provide a gating strategy demonstrating that these are CD11b^+^CD11c^+^ DCs... whether dying cells are PD-1^+^... co-labeling for PD-1, cleaved caspase-3, and CD11c-eYFP."

A full gating strategy (now Figure S5) demonstrate sequential gating from Cells → Doublet → PDPN doublet → CD11b^+^ CD11c ^+^ → CD45IV^+^ (intravascular exclusion positive) within the doublet gate.

(7) (Figures 1F-H): Morphological differences of CD11c^+^ cells.

We have added commentary to the Results section noting that “CD11c^+^ cells in the olfactory bulb parenchyma display a ramified, microglia-like morphology consistent with tissue-resident or parenchymal surveillance cells, whereas those infiltrating the cribriform plate perineural niche show a rounded, non-ramified morphology more consistent with recently recruited monocyte-derived DCs or macrophages.” This morphological distinction aligns with our scRNAseq-defined population differences and supports the notion that the cribriform plate niche shapes distinct myeloid states.

**Reviewer #1 (Recommendations for the authors):**

(1) (Figure 1C): MHCII counts vs. MFI discrepancy

Thank you for catching this. The text has been corrected to reflect that we counted number of cells in the PDPN+ region of the cribriform plate

(2) Proximity ligation assay (PLA) for macrophage-fibroblast ligand-receptor pairs

We appreciate this suggestion. PLA validation of all predicted pairs is beyond the scope of this revision, and we are primarily interested in interactions occurring in vivo and in situ. Future studies will investigate properties of these cells using PLA.

(3) (Figure 2E vs 2G): Inconsistent quantification strategies; CSF1R-GFP/CD11c-eYFP validation of CHI3L3^+^/Arg1^+^ cells

Arg1 signal was more broadly expressed and it was hard to distinguish 1 cell vs 2 cells in close proximity. Which is why we elected to use %Arg1 in PDPN+ regions. Conversely CHI3L3 staining revealed more easily identifiable single cells for quantification. Nonetheless both methods achieve the purpose of outlining that these cells increase in number a the cribriform plate lymphatic regions.

(4) (Figure 3E): Pro-inflammatory features of migratory DCs vs. suppressive interpretation.

"Pdcd1lg2, Cd80, Cd83 are associated with T-cell activation — how does this align with an immunosuppressive niche?"*

This is an excellent point that we now explicitly address in the Discussion. The co-expression of Pdcd1lg2 (PD-L2), Cd80, and Cd83 by migratory DCs likely reflects a tolerogenic activation state rather than a conventional immunostimulatory one. PD-L2 co-expression with costimulatory molecules has been documented in tolerogenic DCs that can engage T cells while simultaneously delivering inhibitory signals via the PD-1/PD-L2 axis (inhibiting rather than amplifying T-cell responses). Furthermore, the lower abundance of migratory DCs in post-contact samples relative to no-contact samples may reflect that cells expressing this immunological synapse machinery are preferentially undergoing programmed cell death (consistent with Figure 6 findings), leaving a post-contact population enriched for the macrophage-dominated tolerogenic signature. We now discuss this interpretation explicitly.

(5) (Figure 5F-G): Gating strategy for PD-1^+^ DCs — PDPN inclusion

The gating strategy has been clarified in Figure S6 (new figure) and the Methods section. PD-1^+^ DCs shown in Figures 5F-G were gated from the PDPN^+^ doublet fraction specifically, paralleling the outlined scRNAseq approach. We have added PDPN as an explicit gate in the updated Figure S6A

(6) (Figure 5H): Discrepancy between text and data — "lowest genes" in PD-1neg DCs.

We apologize for this error. The text has been corrected: the data in Figure 5H show that chemokines, ISGs, and MHC genes are among the highest expressed in PD-1^+^ DCs (not PD-1^-^), consistent with the heatmap shown. This aligns with the interpretation that PD-1^+^ DCs, while tolerogenic, retain antigen-presentation and chemokine-signaling capacity.

(7) Figure 6 reference errors in Results text

Corrected throughout — all references to cell death/apoptosis data now correctly cite Figure 6.

**Reviewer #2 (Public review):**

(1) Sorted populations — in vivo interactions vs. ex vivo aggregation artifacts

As detailed in our response to Reviewer 1 (Weakness 2), due to the non-detectable doublet frequency in non-EAE mice, we believe that PDPN^+^ doublet enrichment is EAE-dependent. We also used cold dissociation conditions. We also note that the transcriptional signatures recovered from PDPN^+^ doublets are not simply a mix of independently sorted PDPN^+^ and myeloid single-cell transcriptomes, they contain unique interaction-associated gene programs (e.g., elevated Pdcd1, tolerogenic markers) not present in non-contact controls, arguing for biologically meaningful contact rather than artifactual aggregation.

(2) PDPN as stromal vs. lymphatic endothelial cells — which is most relevant?

We have clarified throughout the manuscript that PDPN in IHC marks at least two distinct populations at the cribriform plate: (1) PDPN^+^LYVE-1^+^ lymphatic endothelial cells and (2) PDPN^+^LYVE-1^-^ meningeal fibroblasts/perineural sheath cells. It is hard to dissociate which is most relevant in the present study.

(3) Descriptive nature; lack of functional correlates; implications need further discussion.

We appreciate this honest assessment. We agree that functional experiments (e.g., conditional deletion of DC populations at the cribriform plate, blockade of PD-1/PD-L1 axis, lymphatic ablation) will be critical for establishing causality and are ongoing in the laboratory. In this revision, we have:

(1) Added a pseudotime analysis as a computational functional inference.

(2) Refined the Discussion to explore functional implications, including how tolerogenic conditioning at the cribriform plate may limit cervical lymph node priming, parallels with perineural immunosuppression in cancer, and therapeutic opportunities (e.g., modulating this niche to enhance or dampen CNS autoimmunity).

**Reviewer #2 (Recommendations for the authors):**

(1) (Figure 1E): What does PDPN thickness increase represent?

We have added clarification to the Results and Discussion. Based on our data, the increased PDPN^+^ layer thickness during EAE most likely reflects a combination of: (1) increased PDPN expression per cell (supported by elevated MFI in flow cytometry), (2) cellular hypertrophy of existing PDPN^+^ cells. However we cannot fully discriminate between these mechanisms with the current data and acknowledge this as a limitation.

(2) (Figure 2A): In Figure 2A, can the authors provide a healthy control example to pair with 2A? Is the Chi3L3 expression "below" the plate...in the mucosa, associated with EAE, or the same in steady state? The images in 2D are hard to appreciate at the current size.

Healthy (naive) control images are included in Figure 2D for direct comparison with EAE tissue, we added zoomed images of each panel to provide clearer context for the disease-associated changes in myeloid cell distribution and M2 marker expression.

(3) What is the denominator for the quantification in 2E? Is this per unit area? If so, is it the PDPN area or the total cribriform plate region area? If the area of PDPN increases (as the authors show), then the potential area that can hold YM1+ cells also increases, so the absolute number of cells comparison isn't that fair.

We have added this distinction to the results.

(4) The same goes for 2G; however, in G, the quantification is "% Arg1+" ----percentage of what? The increase in Arg1 expression is striking, but it's also striking how similar the PDPN network appears between healthy and EAE in Figure 2F.

We have added this distinction to the results And added a label of quantification to Figure 2G.

(5) Are these increases in Arg1+ cells occurring in the meninges of EAE mice? Or is this specific to perineural areas at the cribriform plate? In a sagittal plane, are these cells clustered tightly at the cribriform plate, or do they extend outward along the ON tracts?

These are clustered tightly in the meningeal regions and along ON tracts. We do not have any sagittal sections available for further proper analysis.

(6) In Figure 2, some panels are labeled "merge" -what does this mean? The DAPI label within the figure is also impossible to see.

Figure labels have been adjusted. Merge is a common label which identifies panels with all channels merged together in a series.

(7) Figure 3: The authors sort cells that interact with PDPN+ CD31+ double-positive cells before the scRNAseq analysis. However, it's not clear from these data that the PDPN expansion observed in their histochemistry is on stromal or endothelial cells. As the authors note, PDPN "also efficiently labels meningeal layers surrounding them along the olfactory nerve layer, including fibroblasts and their associated extracellular matrix (ECM)". Can the authors more clearly explain the rationale for using CD31 in this gating strategy?

We sorted for CD11b+CD45+ (immune), CD31+ (endothelial), PDPN+ (meningeal fibroblasts). CD31 was used to isolate myeloid cells and endothelial cells at the brain’s borders.

(8) Also, without having to do scRNAseq, could the authors compare the interacting populations for cells stuck with PDPN+CD31neg cells? Figure 3B indicates that a good number of these PDPN+CD31neg cells are present in the sort.

We did not isolate PDPN+CD31- cells from our sort, in our experience these are mostly fibroblasts though. Future studies will look at cells which adhere specifically to PDPN+CD31- aggregates.

(9) The interacting cells seem to have a particular affinity for the sorted endothelial cells. However, it's not clear if these cells are simply seizing an opportunity to stick together once the cells are mechanically separated and spun down, or were together in vivo. The authors should determine how many of these cell types are maintaining an in vivo contact or simply are efficient at making new contacts ex vivo. One approach would be to take EAE tissues from CD45.1 and CD45.2 congenic animals and mechanically separate them together. Then the composition of doublets can be analyzed for the frequency of CD45.1/2 doublets or CD45.1 and CD45.2 single positive doublets....and also which cell types are contributing to these doublets. This will test how much of this interaction is driven by ex vivo stickiness or in vivo, and also give some idea about the inherent ability of these immune cells to find and engage PDPN cells.

This is a limitation of the current study, and you have provided an excellent experiment and one we have added to discussion.

(10) Figure 4: I'm confused about Figure 4. If I'm reading this correctly, these are the same data from Figure 3 that were sorted for CD31 positivity. If that's the case, how are there fibroblasts in these data? Does this represent an aggregation of endothelial, fibroblast, AND immune? (CD31, PDPN, and CD11c).

Yes we suspect that endothelial, fibroblast, AND immune aggregates are highly heterogeneous. Without negative sorting/gating we are left with high number of immune cells in or sorting paradigm.

(11) The authors comment on the relatively unclear biological significance of PD1 expression by DCs (non-T cells) and note their previous report on PD1 ligand expression in this cribriform region. Do the authors detect differential PD1 ligand expression in this current study (singlet vs aggregate)?

We have not detected any significant difference in CD274 expression between non-interactor and interactor populations.

(12) Are the FACS data Supplemental Figure 2 on singlet vs doublet DCs performed after Liberase treatment? The FACS plots for both doublet and singlet populations look very different in how they are rendered, with large cell numbers in the 10^-4 range for the doublet groups. Why is this?

No liberase treatment was given in these experiments, we have updated the figure legend.

(13) It seems like the figure labeling has gone awry. On page 9, what should be Figure 5 is being called Figure 4...and further on, Figure 5 is being used for Figure 6 ("Blood derived" data)---this makes it pretty confusing.

This has been corrected. Thank you.

(14) On page 10, the authors have written "Lowest genes in PD-1- DCs included chemokines CXCL9, CXCL10, IL-12b, interferon-stimulated genes (Ifit1, Ifit2 and Ifit3) and several MHC-related genes (H2-M2, H2-Eb2, H2-DMb2)". Is this correct? Based on my reading of the figure, "5H" is that not PD-1+ DCs instead of PD-1- DCs? Also, there is a typo, "Cxck10".

Thank you for pointing this out. We have corrected.

(13) It's not clear what the statement "...these data support that Pdcd1 expression in migratory DCs exhibits an immunosuppressive gene signature..." means. The PD-1 marker cannot "exhibit" anything by itself. Is this intended to say that migratory DCs expressing PD1 exhibit an immunosuppressive phenotype?

Yes this is a better way to say it, it has been corrected.

(14) Figure 6: These are really cool data about the influx of peripherally derived cells to the cribriform plate during EAE. However, it would be more meaningful to have other compartments to compare with. What is the IV+ percentage within the CNS or meninges more generally? And also, how do these CD11c+ CD11b+ aggregates differ in IV+ from "singlets"? The authors show that T cells are caught in the scRNA aggregates. Are these IV+? Can the authors provide additional discussion about the relevance of the Ghost+ data? What does this really mean? In Figure 6, Olfactory is misspelled 2x in A...and the "D" in CD45 is missing from B.

Spelling mistakes have been corrected, thank you. Future investigations will compare IV+ recruitment and aggregations to dural and other brain regions. We suspect that some of the IV+ populations are T cells but our experiments do not allow for this distinction. We have added additional information regarding our interpretation of the Ghost+ data.

(15) The title of the paper indicates that a suppressive myeloid network is assembled, and certainly, there is gene and protein expression data that are consistent with the presence of "suppressive" cells. However, can the authors demonstrate that this "network" is performing a suppressive function in vivo?

This is a great point. Our IHC is highly indicative of classical M2 phenotype accumulating at meningeal regions around the olfactory bulb. One experiment we are interested in is local ablation of macrophages at the CP, to determine their role in EAE disease progression.

(16) At the end of the discussion, the authors state, "They describe unique DC populations at the cribriform plate, one displaying pro-inflammatory and migratory features while the PDPN-associated population displayed more immunoregulatory characteristics". This seems a little bit misleading, or at least not giving the macrophages their due. A good part of the migratory DCs (as put in the figures) are associated with the Arg1+/Chi3l3+ macrophages. It's possible that suppression -if it's happening- could come from one or both cell types.

We have removed that line and altered the discussion to more accurately reflect the results with respect to DCs and Macrophages.

(17) In this study, the authors focus on dendritic cell and macrophage populations in the context of autoimmune disease and chronic CNS inflammation. In a recent study, the authors show an important recruitment of immune cells in the cribriform plate during a CNS infection by *Mycobacterium tuberculosis*. Do Arg1+/Chil3l3+ macrophage and tolerogenic DC populations still exist in this context? It would significantly strengthen the field's understanding of how the cells of the cribriform behave in different conditions if you could describe whether these cells are context-specific or is it really specific to cribriform plate tissue?

This is an excellent suggestion and will be the focus of future investigations.

We believe these revisions substantially strengthen the manuscript and directly address major concerns raised by both reviewers. We remain committed to the functional follow-up studies that both reviewers rightly identify as the natural next chapter of this work.
